# In the West Indies

**Published:** 1881-09

**Authors:** 


### IN THE WEST INDIES.

Strange as it may seem at first sight, everybody in the West Indias eats very large meals. The climate is so hot that you take food freely to make up for the losses, and the appetite has to be stimulated by a great variety of dishes, as well as by the copious use of those very insidious capsicums, and the still more delicious little red and yellow bird-peppers. A few of these tempting fruits are placed in the salt-cellars at every meal, and with the bright tropical flowers which invariably garnish the table in pretty specimen vases, they give a general air of pleasant æsthetic refinement to the whole arrangements.. Breakfast is a really solid and substantial repast, usually put off till half past ten or eleven o'clock, the pangs of pressing hunger being stilled before the early morning canter by a cup of coffee in the bedroom. With it sometimes comes a cassava-cake, one of the best Jamaican institutions, made by the negro villagers from the roughly-scraped meal of the arrow-root plant. This meal is rolled into a thin paste and then baked hard and dry into round cakes about the thickness of a Scotch oatmeal bannock, but much more delicate in taste.—*Belgravia.*

To Relieve Hiccough.—A medical journal gives the following simple means of relieving hiccough: Inflate the lungs as fully as possible, and then press firmly on the agitated diaphragm. In a few seconds the spasmodic action of the muscle will cease.

